# On characterizing protein spatial clusters with correlation approaches

**DOI:** 10.1038/srep31164

**Published:** 2016-08-10

**Authors:** Arun Shivanandan, Jayakrishnan Unnikrishnan, Aleksandra Radenovic

**Affiliations:** 1 Laboratory of Nanoscale Biology, Institute of Bioengineering, Ecole Polytechnique Federale de Lausanne (EPFL), Lausanne 1015, Switzerland; 2 Audiovisual Communications Laboratory, School of Computer and Communication Sciences, Ecole Polytechnique Federale de Lausanne (EPFL), Lausanne 1015, Switzerland

## Abstract

Spatial aggregation of proteins might have functional importance, e.g., in signaling, and nano-imaging can be used to study them. Such studies require accurate characterization of clusters based on noisy data. A set of spatial correlation approaches free of underlying cluster processes and input parameters have been widely used for this purpose. They include the radius of maximal aggregation *r*_*a*_ obtained from Ripley’s *L*(*r*) − *r* function as an estimator of cluster size, and the estimation of various cluster parameters based on an exponential model of the Pair Correlation Function(PCF). While convenient, the accuracy of these methods is not clear: e.g., does it depend on how the molecules are distributed within the clusters, or on cluster parameters? We analyze these methods for a variety of cluster models. We find that *r*_*a*_ relates to true cluster size by a factor that is nonlinearly dependent on parameters and that can be arbitrarily large. For the PCF method, for the models analyzed, we obtain linear relationships between the estimators and true parameters, and the estimators were found to be within ±100% of true parameters, depending on the model. Our results, based on an extendable general framework, point to the need for caution in applying these methods.

In cell biology and elsewhere, spatial aggregation or clustering is an interesting phenomenon, possibly with a functional role — e.g., the behavior of proteins to form sub-micrometer sized clusters could be important for their functionality, e.g. in signaling^1^,^2^,^3^,^4^, transcription^5^,^6^, etc. The origins, structure and function of spatial heterogeneity of proteins in varous systems are only being studied. Spatial location information, available from fluorescence and electron microscopic imaging, and recently from sub-diffraction limited fluorescence imaging such as Single Molecule Localization Microscopy(SMLM) techniques like Photo-Activated Localization Microscopy (PALM) and STochastic Optical Reconstruction Microscopy(STORM)^7^,^8^,^9^, are key to such studies^10^,^11^,^12^,^13^. Accurate characterization of clustering — its strength, scale and density — is an important part of these studies, whether for relative comparison between different systems, perturbation conditions and to test hypothesis (e.g. the relative importance of lipid rafts and actin cytoskeleton in membrane protein clustering^14^, or the possible mechanisms of early T-cell signaling^10^), or for absolute quantification, e.g. the size of clusters in a particular cell type in a particular condition, and the number of molecules in them.

A number of methods have been used to characterize the clusters from imaging data^15^,^16^,^17^. The methods can be broadly categorized into two: (1) clustering or segmentation to identify the clusters, followed by their characterization; and (2) spatial statistics approaches based on a second-order spatial summary statistic like Besag *L*(*r*) − *r* function or the Pair Correlation Function *g*(*r*). These second-order functions can be used for comparison of clustering at different scales and between different experimental systems and perturbations, and estimators based on these functions can be used for ensemble cluster parameter estimation. In general, they have a few advantages over many of the segmentation approaches: they are parameter-free, can detect interactions at multiple spatial scales, can work with both dense and sparse point patterns, often have direct physical interpretations^18^, and are amenable to rigorous extensions incorporating error models, crucial in the case of nanoscale imaging^18^,^19^,^20^. Specifically, models of various sources of errors in SM imaging, such as the artifact clustering due to single fluorophore blinking^11^,^18^, localization uncertainty of 10–50 nm (FWHM) due to limited number of signal photons collected and the imperfect detection efficiency (only 40–60% of photo-activable fluorophores in the sample can be typically detected) can be incorporated to correlation functions^11^,^18^,^20^. Also, in the case of SMLM, the notion of spatial point patterns align well with the nature of its point localization readout. In practice, a major convenience of using such methods have been that they estimate ensemble functions at different scales and the various cluster parameters for a whole dataset, without requiring user set input parameters, making comparative studies easy in systems where variability within cluster sizes are not important.

Two spatial statistics based estimators of cluster parameters based on these functions widely reported in the nanoimaging and protein cluster analysis literature are 1) the radius of maximal aggregation *r*_*a*_^15^,^17^,^20^,^21^,^22^,^23^,^24^,^25^,^26^,^27^,^28^,^29^, the radius value corresponding to the maxima of the empirical *L*(*r*) − *r* function, as an estimator of cluster size(length scale); and 2) the functional approximation of *g*(*r*) as an exponential function^5^,^11^,^18^,^30^,^31^,^32^,^33^, extended to 3D in ref. 34, leading to estimators of cluster size, amplitude or strength and number of molecules per cluster (see Methods). While both methods are based on second-order correlation, the estimators are different – one is based on the empirical maxima of the *L*(*r*) − *r* function while the other is based on fitting the empirical pair correlation function to an approximate model to obtain the model parameters. These methods are not concerned with the underlying spatial distributions, e.g. the shapes of clusters and the distribution of molecules in them. Effects due to differences in underlying spatial distribution are either ignored or approximated, making the estimation process free of underlying cluster processes.

However, molecular distribution in clusters observed through bio-imaging could be of different shapes, depending on the underlying physical mechanism. The molecules could be concentrated at the center of the cluster, either heavily or lightly, or distributed uniformly within the cluster (Fig. 1). In the case of SMLM imaging, e.g., the clusters formed due to photoblinking are reported to have a Gaussian^11^ or Cauchy peak shape^33^ – the latter a well known “fat tailed” distribution – depending on the photon count distribution within the cluster. It is plausible to model internalization in circular or spherical bodies with a hard-core process (a disk in 2D). Liquid clusters, due to surface tension, are expected to form spherical clusters^35^. Analysis methods often assume Gaussian shapes for membrane clusters^16^,^17^. Refs 36,37 have suggested modeling membrane protein distributions using 2D-Ising model, to account for phase transitions and criticality. It is not clear how the parameter estimation approaches that are independent of underlying true cluster processes are biased or scaled due to these different underlying molecular distributions in clusters. They also raise the question of identifiability: e.g., can the size (i.e. length scale) estimator of these approaches be mapped exclusively to the size parameter of the true process, independent of other parameters, such as number of clusters per unit area or cluster density or amplitude? If the estimated size parameter is dependent on both the size and amplitude parameters of the underlying true process, one must account for it during the comparative analysis of cluster sizes, as it may not accurately reflect the true differences in size, estimation being affected by amplitudes as well. Other point pattern based parametric methods^26^,^38^ also have to deal with similar issues. The influence of shape and geometry – of crucial importance in biology^39^ – in estimation is observed in other fluorescence based technologies as well^40^.

Some clues have been obtained from simulation studies. Kiskowski *et al.*^21^ studied the relation between the true radius of disk clusters *R* and estimates of *r*_*a*_ by means of simulations, and derived important insights — such as *R* ≤ *r*_*a*_ ≤ 2*R*, and a dependency of *r*_*a*_ on separation between clusters. However, since the study was based on simulations, with a limited set of parameters and models (only disk clusters), the understanding is limited, and the possibilities of generalization are not clear. Lagache *et al.*^26^ performed a theoretical analysis of a similar estimator — maxima of the *K*-function normalized with its variance — for disk shaped clusters, and reported a simpler, constant relation *r*_*a*_/*R* = 1.3. Such a relationship would have been convenient, however its generality in terms of models and parameters is not clear. No studies of the bias introduced by the approximate model of *g*(*r*) has yet been reported, to the best of our knowledge.

Note that the accuracy or bias of an estimator cannot be improved by repeated measurements, unlike its precision. By definition, bias affects absolute quantification. The same is the case regarding their use as relative comparisons: the need to account for biases might become important if (1) the parameters are not separately identifiable or (2) involves scaling that are model dependent and the model is not known, or if the comparisons involve different models.

In this work, we explore, with theoretical rigor, the bias in parameter estimation and the questions of identifiability introduced by these approaches (Fig. 1). We consider a number of spatial cluster processes whose theoretical *g*(*r*) and *L*(*r*) − *r* are known, and then derive the relation between the parameters of these approaches (such as *r*_*a*_) and the true process parameters (e.g., the cluster size parameter *r*_*t*_). We find that, in general, for a large class of clustered point patterns, the ratio *p* of the radius of maximal aggregation *r*_*a*_ and the size parameter of the true process *r*_*t*_(*p* = *r*_*a*_/*r*_*t*_) can be derived as an implicit nonlinear function of two cluster parameters: *r*_*t*_ and the number of clusters per unit area (*κ*). We also find that it possible to derive a theoretical lower bound for *p*, given a cluster model following some basic assumptions. We validate the theoretical results with simulations. We also perform similar analysis for the normalized *K*-function presented in ref. 26, to report a more complex relationship between the true cluster size and the estimator than presented in ref. 26. Then, we investigate the bias due to the exponential approximation model of *g*(*r*), for the different models. By minimizing the Least Square Error between the true and approximate PCFs, we obtain scaling laws between the approximate model and the true model parameters, and validate the approach by simulations. The extension for other cluster models is straightforward.

## Results

### Parameter identifiability issues and bias of radius of maximal aggregation

Here we obtain the relationship between the radius of maximal aggregation, defined as 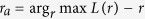, and the true cluster size parameter (detailed definitions in Methods). We focus on a class of clustered point patterns with *K*-functions of the form


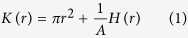


with *h*(*r*) = *H*′(*r*) and *A* > 0. Such a shape for *K*-function can represent diverse cluster models, such as uniform disk, Gaussian, etc (called Poisson cluster processes) and Ising processes (see Methods, Supplementary Table S1, for details). *H* typically is a function of the true cluster size parameter. The parameter *A* represents the number of clusters per unit area (*κ*) in the case of Poisson cluster processes, and ‘amplitude’ in the case of the Ising model (Methods). *L*(*r*)−*r* is typically also used to compare the ‘strength’ of clustering, and the expressions in Supplementary Table S1 relates *L*(*r*)−*r* to different cluster parameters. It can be noted that for a large class of models, the expressions of *L*(*r*)−*r* are independent of the number of molecules per cluster (Methods).

For point processes with the *K*-function as in (1), using the basic criteria for local maxima, we obtain *L*′(*r*_*a*_) − 1 = 0, and hence *K*′(*r*_*a*_)^2^ = 4*πK*(*r*_*a*_) using (5). Substituting this in (1), we obtain


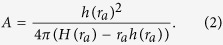


That is, *r*_*a*_ depends on *A* in general, as *A* is not a parameter of *H* and *h*. (2) can be used to obtain a relation between 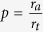 for all the models listed in Supplementary Table S1, where *r*_*t*_ is the cluster size parameter of the true process. The results are shown in Supplementary Table S2 and the details of derivation are given in Supplementary Information.

**Figure 2:**
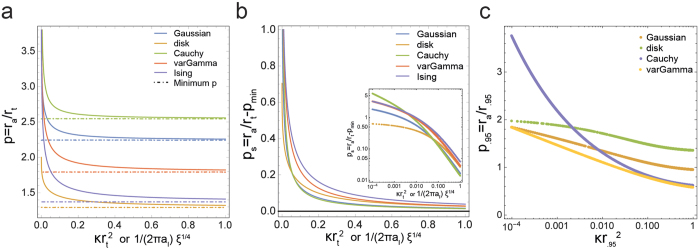
Relation between the radius of maximal aggregation and true cluster size. (**a)** For different cluster models, the relation between the ratio of radius of maximal aggregation *r*_*a*_ and cluster size parameter of the true process *r*_*t*_, as a function of the number of clusters per unit area *κ* and *r*_*t*_. The minimum *p* value is obtained by exploiting the singularity in (2), also listed in Supplementary Table S2 (**b)** Plots in (**a)** after translating by the minimum *p* and in log-log scale (inset). Note the partial power law like shape. (**c)** *p*_0.95_, the ratio between *r*_*a*_ and *r*_0.95_, the latter being the true scale within which 95% of all clustered points lie, plotted against 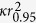. It can be seen that the relationships are model dependent. Note that for a sample with 10 clusters per *μm*^2^ and *r*_0.95_ = 20 *nm*, 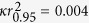.

We find that it is possible to write the relationship 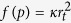 for all the Poisson cluster processes discussed, and hence, *p* (and *r*_*a*_) is non-linearly dependent on both *κ* and *r*_*t*_ (Supplementary Table S2). In the case of exponential PCF considered in detail in the next section, of which the varGamma process is an example, the results – non-linear relation between *r*_*a*_, *r*_*t*_ and *κ*, hold as well. In the case of the Ising process, the corresponding relationship is of the form 
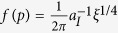
, where *a*_*I*_ and *ξ* are the amplitude and size parameters of Ising process (Methods, Supplementary Tables S1). Figure 2a shows the contour plot of *p vs κr*_*t*_ for different models. The behavior of *p* in the case of a power-law PCF — heavily dependent on parameters — is given in Supplementary Figure S5 (derivation in Supplementary Information). Note that *p* is independent of the number of points per cluster (*μ* in Methods) if the expressions for *K*-functions are independent of it.

The singularity at *H*(*r*_*a*_) − *r*_*a*_*h*(*r*_*a*_) = 0 in (2) provides a lower bound for *p* for all the models analyzed, and is also shown in Supplementary Table S2 and Fig. 2a. The lower bound so obtained is a fundamental characteristic of the cluster model. The existence of a lower bound for *r*_*a*_ for any cluster model with *K*-function of the form in (1) can be proved theoretically given some basic assumptions on *h*(*r*) (see Supplementary Information).

It can be seen that the lower bound for *p* is model dependent. For the disk model, e.g., 1.29564 < *p* < 2, whereas, for Gaussian model, 2.24181 < *p* < ∞. Now, the *p* for different processes cannot be directly compared, as the size parameter of the true process *r*_*t*_ is defined differently for them. A more comparable measure would be *r*_*q*_, the (true) radius at which *q* fraction of the points are expected to lie for a particular distribution, typically obtainable in the form *r*_*q*_ = *u*_*q*_*r*_*t*_, such as the case of *r*_0.95_ = 2*σ* in the case of 1D Gaussian distribution. *r*_*q*_ is conceptually similar to “full width at half maximum” (FWHM), a measure that is widely used in the imaging literature. *r*_q_ would then correspond to the ratio *p*_*q*_ = *p*/*u*_*q*_. Considering the case *q* = 95%, the values for *u*_0.95_ and the lower bounds for *p*_0.95_ for different distributions is given in Supplementary Information, and the plot *p*_0.95_ *vs* 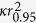 is shown in Fig. 2c. It can be seen that *p*_0.95_ is dependent on both the model as well as both the number of clusters per unit area and the true cluster size.

The systematic relationship established between *p* (or *p*_0.95_), *A* and *r*_*t*_, clarifies the bias and identifiability issues in estimation. The results agree with ref. 21, and provide a tighter theoretical lower bound (1.29564 instead of 1) for disk clusters. The approach can also explain the qualitative influence of inter-cluster distance on *r*_*a*_ observed by^21^, through the dependency of *p* on *κ*, *r_q_* would then correspond to the ratio. The dependency of *p* on other cluster parameters and the cluster model means that the estimator could be a poor choice as a comparison tool between different experiments *r_q_* would then correspond to the ratio.

### Validation with simulations

To establish the validity of the theoretical derivation obtained in previous section (shown in Supplementary Table S2) we performed a Monte Carlo simulation study. In addition to information about the accuracy of radius of maximal aggregation (the subject of the theoretical study), it also provides information about its precision as an estimator.

**Figure 3:**
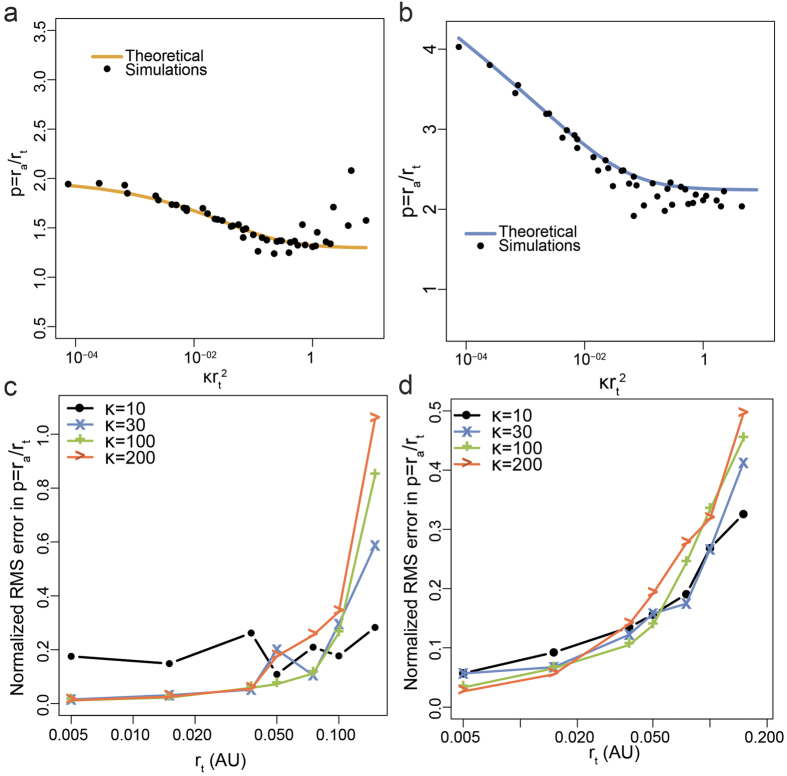
Comparison of theoretical results on *p* = *r*_*a*_/*r*_*t*_ with that from simulations. (**a**,**b)** Results from theory (solid curve) as well as simulations on unit square window (dots), for disk and Gaussian clusters respectively. Only the mean value from 100 simulations are shown, for clarity, and the plot with error bars can be seen in Supplementary Figure S1. It can be seen that in both disk and Gaussian cases, the mean values from simulations deviate from the theoretical values with increasing 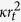. (**c**,**d)** The Root Mean Squared error, normalized by the theoretical value, for disk and Gaussian clusters respectively, plotted against *r*_*t*_. The colors denote different *κ* values. It can be seen that the error values are highly influenced by *r*_*t*_.

Clustered point patterns, belonging to either Gaussian or disk clusters, were simulated in a unit square, for varying *κ* and *r*_*t*_. The theoretical value of *p* for a given *κ* and *r*_*t*_ were obtained by solving the analytical expressions in Supplementary Table S2, and was compared to 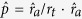 was obtained from the empirical maximum of the *L*(*r*) − *r* curves. The results are shown in Fig. 3 (also see Supplementary Figure S1). The mean value of 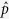 from simulations broadly agree with the theoretical results, though the deviation increases with increasing 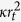 (see also the Mean Squared Error in Fig. 3c,d). This is probably the result of increasing number of clusters per unit area (increasing *κ*) or having larger clusters within the unit square used in the simulations (increasing *r*_*t*_), both resulting in overlapping clusters, resulting in deviations from theoretical framework based on a particular cluster model. In fact, it can be seen that the deviation is most influenced by increasing radius (Fig. 3c,d).

### Case of normalized *K*-function

In ref. 26, a variation of *K*-function was introduced, referred to as 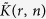 (details in Methods). The radius of maximal aggregation 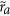 for 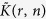 was then obtained by setting 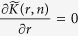. Using numerical approaches, they obtained the constant relation 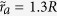.

**Figure 4:**
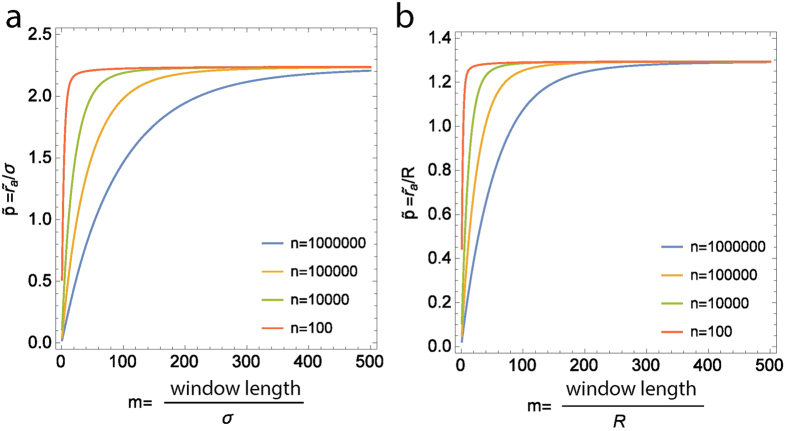
Results in the case of normalized *K*-function 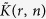 (**a)** Gaussian clusters, (**b)** disk clusters. In the case of 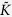, the 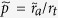 depends on the number of points *n* and the ratio *m* between the size of the observation window (side length of a square in this case) and the true size parameter *r*_*t*_, and converges to a maximum value at large *m*, which is approximately equal to the minimum values obtained in the case of *r*_*a*_ based on *L*(*r*) − *r*. Note that in the case of clusters with *R* = 20 *nm* with an area of analysis of size 10 *μm*, *m* = 500, and 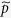 is close to the maximum value, and hence a constant. On the other hand, if the area of analysis was smaller, say 1 *μm*, *m* = 50, and 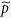 depends on *n*.

In our hands, 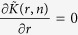 for a square observation window (for simplicity) resulted in a more nuanced situation, as shown in Fig. 4 (details in Supplementary Information). We found that 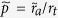 depends on the number of points *n* and the ratio *m* between the side length of the square observation window and the true size parameter *r*_*t*_, and converges to a maximum value (an upper bound) at large *m*, which is approximately equal to the minimum values obtained in the case of *r*_*a*_ based on *L*(*r*) − *r*. For example, in the case of clusters with *R* = 20 *nm* with an area of analysis of size 10 *μm*, then *m* = 500, and 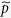 is close to the maximum value (Fig. 4), and hence a constant (1.296 in the case of disk clusters, approximately equal to the factor of 1.3 obtained in ref. 26). On the other hand, if the area of analysis was smaller, say 1 *μm*, then *m* = 50, and 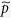 depends critically on *n* (Fig. 4). The dependency of 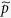 on *n*, in contrast with *p* in the case of *L*(*r*) − *r*, is because 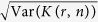 used in the definition of 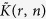 (Methods) is non-linearly dependent on *n*, whereas the expression for *K*(*r*, *n*) (and *L*(*r*) − *r*) is independent of *n*. Note that 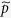 is independent of *κ* and *β*, unlike the case of *r*_*a*_ and *L*(*r*) − *r* presented in the previous section.

### Estimation based on exponential approximation of Pair Correlation Function

**Figure 5:**
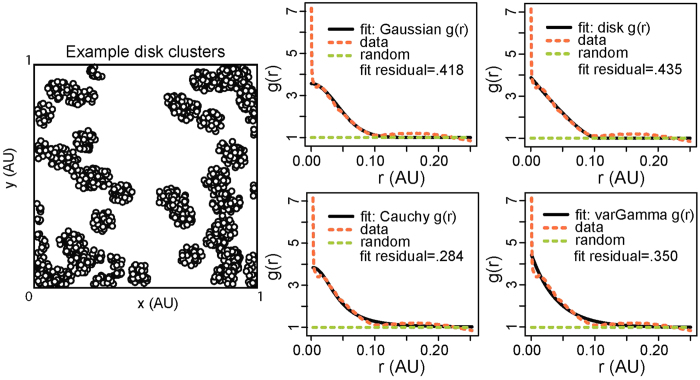
Demonstrative example of fitting model PCFs to the empirical PCF of a disk point pattern. The empirical PCF of the point pattern in the left is calculated, and is fit to the theoretical PCFs of various cluster processes. *g*(*r*) denotes the PCF. Fit results 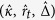, 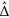 being the value of the objective function for the best fit parameters, called fit residual: Gaussian (38.11, 0.028, 0.418), disk (40.64, 0.052, 0.435), Cauchy (21.55, 0.051, 0.284), varGamma (27.86, 0.040, 0.350)), whereas the true values of the disk point pattern are (*κ* = 50, *r*_*t*_ = *R* = 0.05). Note that 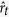 is defined differently for different processes (Supplementary Table S1). The Cauchy distribution is found to have the best fitness, whereas the disk one — the true model — has the worst. The *p* = *r*_*a*_/*r*_*t*_ corresponding to disk distribution, with the estimated parameters above is 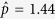. The maxima of *L*(*r*) − *r* is at 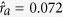, providing a 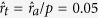, equal to the true *R*.

We now consider another estimator that has been suggested for estimating cluster parameters, the approach based on fitting Pair Correlation Functions. As discussed in the section Methods, the theoretical PCF is not unique to a cluster model, and its signature shape and sensitivity are often not sufficient to identify the models (e.g., see Fig. 5), not the least because each experiment provides a realization of a stochastic process, with the observed statistic approaching the theoretical one only as *n* → ∞. Model selection based on Monte Carlo (MC) rank tests^41^,^42^ — ranking the empirical statistic value among the values of the statistic from MC simulations based on estimated parameters — based on PCF or the related *K* or *L*(*r*) − *r* functions is not sound, if the same function was used for parameter estimation^41^. The standard method in this case is to perform MC rank tests with a statistic that is different from the one that was used for parameter estimation, e.g., the nearest neighbor distribution function if the PCF was used for estimation. However, the approach is known to have low statistical power^42^, and we too had similar experience during preliminary attempts to identify the cluster models from simulations and SMLM data (results not shown). Therefore, functional approximations such as *g*_*a*_(*r*) = 1 + *a* exp(−*r*/*d*), proposed as part as the PC-PALM method, have much appeal.

Here, we derive a measure of bias in parameters introduced by this approximation, given a true model. We aim to find the relations *m* = *d*/*r*_*t*_, *n* = *a*/*a*_*t*_ and *l* = *N*_*a*_/*N*_*t*_, given a true model for the PCF in the form *f*(*r*) = 1 + *a*_*t*_*v*(*r*, *r*_*t*_). Here, *N*_*a*_ and *N*_*t*_ are the average number of points per cluster corresponding to the approximate model and the true model respectively, as per (7). Given a specific model for *f*(*r*), we find the relation between parameters in the case of the fit that provides the minimum (Least) Squared Error *E*, i.e.,


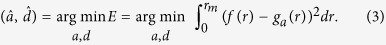


Note that the Least Squares criteria was used in original PC-PALM papers for parameter estimation^11^,^43^. If *E* has a minima at 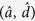, then 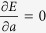 and 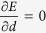 at 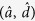, which can be solved to obtain expressions for 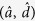. Measures of *m*, *n* and *l* can then be found using these.

**Figure 6:**
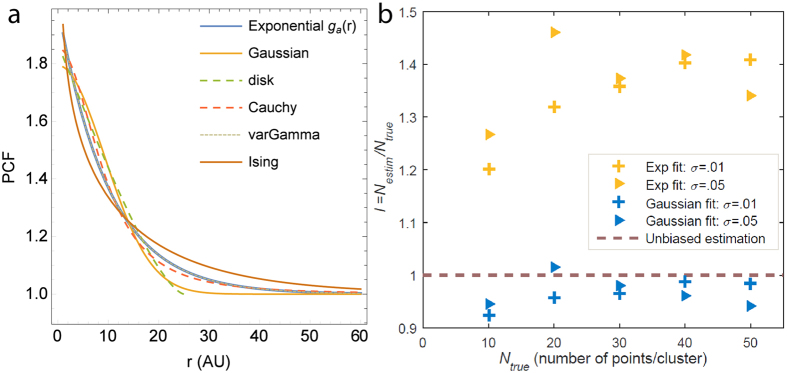
(**a)** Optimal Least Square Error fits for different models. For parameter values *a* = 1 and *d* = 10, the PCFs corresponding to different models in Supplementary Table S1 is plotted, with the parameters scaled as per Table 1. For simplicity, only *r* ≥ 1 is shown. (**b)** Mean estimates of *N* (number of points per cluster) from fitting the empirical PCF of Gaussian clustered point patterns with (1) Gaussian PCF (2) the exponential approximation *g*_*a*_(*r*) (results from 20 simulations on a unit square window). The results broadly agree with the theoretical prediction of *l* = 1.48, approaching it with larger *N*_*true*_. A plot with error bars can be seen in Supplementary Figure S5.

We were able to obtain measures of *m*,*n* and *l* for all the cluster models described in Supplementary Table S1, and the results are shown in Table 1 and the best fit PCFs can be seen in Fig. 6a (details in Supplementary Information). The *m*_0.95_ values: *m*_0.95_ = *d*/*r*_0.95_ = *m*/*u*_0.95_, *r*_0.95_ being the scale at which 95% of points are expected to lie, can also be obtained as constant scalar values, given by 0.63, 0.82, 0.38 and 0.28, for Gaussian, disk, Cauchy and varGamma models respectively.

For example, in the case of Gaussian shaped clusters, with the PCF given in Supplementary Table S1, we obtain, for *r*_*m*_ > 6*σ*, *m* = *d*/*σ* ≈ 1.54, *n* ≈ 1.26, *l* ≈ 1.48, with *m*_0.95_ = 0.63. The parameters can be either *upscaled* or *downscaled* — e.g., the number of molecules per cluster is overestimated by 50% by using *g*_*a*_(*r*) for estimation, whereas in the case of Ising process, it is underestimated by 40%. The overestimation/underestimation for all parameters is no more than by 100% in all the models the approach was applied, except in the case of the amplitude parameter in the Ising model. In this case too, while the *a* parameter is dependent on both the true amplitude *a*_*I*_ as well as true size parameter *ξ*, the effect is to the extend of *n* = 0.38–1.44 for *ξ* = 5–1000 nm, the case relevant in the case of protein clusters.

For the models in Supplementary Table S1, this means that (1) the estimated parameters scale linearly with the true ones, (2) the scaling is either independent of other parameters or only mildly dependent, (3) the theoretical scaling due to the exponential approximation is within 100%, in contrast with the radius of maximal aggregation, which can be several times higher (technically upto ∞) depending on models and parameter values.

We validated this theoretical approach by means of Monte Carlo simulations. We simulated Gaussian cluster processes in a unit square for different conditions, such as varying the numbers of points per cluster as well as cluster radius. The empirical PCF of these point patterns were fitted to both the theoretical PCF for Gaussian point patterns, as well as the functional approximation *g*_*a*_(*r*), and the various parameters estimated. The estimates for *N*, the number of points per cluster is shown in Fig. 6b. It can be seen that the simulations agree with the theoretical prediction, with estimates using *g*_*a*_(*r*) being overestimated, whereas the fit to Gaussian PCF providing accurate results.

## Discussion

Quantitative studies of protein clustering from noisy single molecule data requires accurate and precise estimation tools. A key source of inaccuracy or bias in quantification could be the bias in the estimation tools themselves. Unlike in the case of precision, repeated measurements cannot remove the errors due to bias. The commonly used correlation-based estimators provide a convenient estimation tool by incorporating error models relevant to SM imaging and by not requiring the user to input parameters. However, the results should be interpreted after accounting for estimator bias.

The description of protein clustering involves multiple independent parameters, such as the number of clusters per unit area, cluster length scale, number of molecules per cluster and the cluster shape or distribution, all of which are unknown. Correlation approaches have been used in two ways: (1) to estimate some of these parameters, and (2) to estimate and compare the level or ‘strength’ of clustering between different conditions, e.g., based on the magnitude of *L*(*r*) − *r* curves. The latter task involves estimating a lumped measure that combines the above mentioned parameters, which describes the ‘strength’ of clustering. However, it is not clear how these estimators relate to true parameters. Can the true parameters be estimated independently (identifiability), or do the estimators correspond to some lumped parameters that combine independent true parameters? Also, what does the ‘strength’ of clustering mean with respect to the independent cluster parameters? It might be possible to have the same strength estimate values (e.g., *L*(*r*) − *r* magnitude) for different combinations of parameter values, if their net effect on the lumped measure is the same.

Based on the theoretical correlation functions corresponding to a diverse set of models (Table S1), we have theoretically analyzed three correlation based methods for estimating cluster parameters that have been proposed in the literature, for their identifiability and bias. The results also provide information about how different parameters influence the correlation functions for different cluster processes, i.e., what does ‘strength’ of clustering mean. Our results provide a cautionary tale about using these approaches, and also provides a general framework to analyze their bias.

Specifically, we analyzed: the radius of maximal aggregation based on *L*(*r*) − *r* function and on normalized *K*-function, both primarily estimators for cluster size (length scale), and the estimation based on the functional approximation with an exponential function for the Pair Correlation Function, proposed in the PC-PALM method. The expressions in Table S1 provides the relation between cluster parameters and correlation functions (i.e., strength of clustering). We find that crucial independent parameters of clustering, such as the number of molecules per cluster, may not be reflected in the *L*(*r*) − *r* curves (Table S1). We were able to derive the theoretical relation between the radius of maximal aggregation and the true model parameters, for different models. These relations give an operational interpretation of the radius of maximal aggregation for different cluster generating models. In addition to the relation to model specific size parameter (such as radius for disk clusters, standard deviation for Gaussian clusters, etc), we also analyzed the relation between the radius of maximal aggregation and a cluster size measure that is defined independent of models (radius at which 95% of molecules are expected to lie). Our results illustrate that the ratio of radius of maximal aggregation (from *L*(*r*) − *r* curves) to the true cluster size nonlinearly depends on the true cluster size as well as the number of clusters per unit area (or corresponding parameters, such as amplitude) for all the models considered. While we were able to derive theoretical lower bounds for this ratio, we found that the ratio could be arbitrarily large depending on the parameters and models, illustrating that the radius of maximal aggregation is not always indicative of the true cluster size.

In the case of the approach based on the exponential approximation of Pair Correlation Function, we were able to derive the scaling laws between the parameters of the approximate model and the true model, based on the Least Squares criteria. It was found that the relationship between estimates and true parameters were found to be linear, and that the bias is limited to ±100% of true parameter values for the models we considered. These results apply to model independent measures such as number of molecules per cluster.

Our results provide a cautionary tale in the quantification of clustering based on commonly used correlation approaches. The estimates obtained are crucially dependent on various cluster parameters or models, meaning that considering them as absolute measures might be misleading. Moreover, in the case of many estimators (e.g., *r*_*a*_), a qualitative, monotonic mapping between the corresponding parameters (size) are found to be not possible in general, instead, they might depend on other parameters as well (e.g., number of clusters per unit area *κ* in the case of *r*_*a*_). That is, for certain combinations of parametric values, a higher *r*_*a*_ might not mean a higher true cluster size, since they might be confounded by *κ*. However, there are regimes of parametric values where the dependency on other parameters are not key (see Fig. 2), and guidance for whether the analysis do not fall in such regimes where a qualitative mapping is possible, can be obtained using the expressions in Table S2 (and corresponding figures in Fig. 2) along with estimates of relevant parameters. Since the results in the case of exponential approximation of PCF points to a linear mapping between the parameters and estimators for the models analyzed, and since qualitative, monotonic mapping is possible, it appears to be in general better estimator than *r*_*a*_, for the models analyzed. Unfortunately, there seems to be a significant effect of model dependent scaling, and whether it is an acceptable level of bias depends on the specific problem and the magnitudes involved, and caution must be applied during analysis. Also, we find that the identification of cluster models based on second-order functions is difficult, and we also describe how background noise might crucially affect estimation (Methods). We recommend that the results using the correlation approaches must ideally be corroborated by means of other methods (such as DBSCAN, or recent approaches such as^16^,^17^), especially if the effect size is small.

Although only a limited set of models were analyzed here, the results illustrate the limits of the estimators. The analysis presented here can also be extended to other models and settings in a straightforward manner. For instance, we focused solely on the case of constant cluster size for all models for the purpose of illustration. However, the analysis can be easily extended to variable cluster sizes by modeling the cluster size as a random variable in (10). Also, our analysis shows that it might be possible to obtain theoretical bounds for parameters given a set of candidate models, e.g. by taking the worst bounds among candidates, even though the specific candidate model for a system is not known or is difficult to be inferred. It also points to a possible approach to reducing the bias: by using non-parametric models for the PCF, although care must be taken against overfitting and also in interpreting the results. This work only deals with the accuracy limits of the estimators, their precision could also be important in practical applications, which must be analyzed separately. The results presented in this work are not limited to protein clusters, and are applicable to any system with spatial clustering.

## Methods

### Background definitions

For a spatial point pattern in 2D-space, Ripley’s *K*-function is defined^41^,^42^,^44^ as


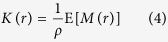


where *ρ* is the spatial density (average number of points per unit area), and *M*(*r*) is the number of other events within distance *r* of a randomly chosen event. The Besag *L*(*r*) − *r*, a measure of cluster strength at *r*, is then given by


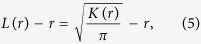


and the Pair Correlation Function by


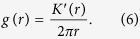


Alternative but equivalent definitions of PCF starting with the notion of spatial autocorrelation are also possible^11^.

The radius of maximal aggregation, 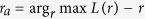.

The function *g*_*a*_(*r*) = 1 + *a* exp(−*r*/*d*) has been proposed as a functional approximation for the Pair Correlation Function (PCF) of “2D-system of clusters with no predefined shape”^11^,^30^. The parameter *a* is the amplitude, a measure of point density in the clusters, and *d*, the correlation length, gives the radius of the cluster^11^. For the PCF *g*(*r*), the average number of points per cluster can then be obtained as


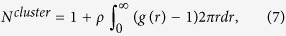


which is equal to *N*_*a*_ = 2*πad*^2^*ρ* in the case of *g*_*a*_(*r*), where *ρ* is the average density of points in the area of analysis^11^.

In ref. 26, the normalized statistic 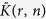 was proposed, given by 
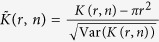
, where


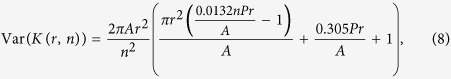


where *A* is the area and *P* the perimeter of the observation window, and *n* the number of points.

For disk process, they use, similar to the expression in Supplementary Table S1:


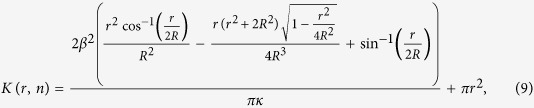


where *κ* is the number of clusters per unit area, and *β* the clustered fraction.

### Theoretical expressions for *g*(*r*) and *L*(*r*) − *r*

In order to derive the theoretical expressions for *L*(*r*) − *r* and *g*(*r*) for different cluster models, it is useful to focus on a class of spatial cluster processes, known as Poisson cluster processes, or Neyman-Scott processes(details in refs 41 and 42), which are generated in the following way. First, a set of *parent* points are created, following a spatial Poisson process (complete spatial randomness) with density (intensity) *κ*. Then, *S* number of points are distributed around each *parent* point according to the i.i.d bivariate PDF *f*_*pdf*_ (.), *S* following some i.i.d distribution with mean *μ*. We assume a constant cluster size (length scale) parameter here, though the analysis with a variable cluster size is straightforward. These *offspring* points form the clustered point pattern. Such simple spatial cluster models that consider different shapes of clusters provide a starting point for the theoretical analysis of estimators. The Ising model, also considered in analysis, provides a more physical example.

Assuming *f*_*pdf*_ (.) to be radially symmetric, let the PDF of the distance *r* between two offspring points within a cluster is given by *h*_*d*_(*r*) and its Cumulative Distribution Function (CDF) by *H*_*d*_(*r*). Then^41^:


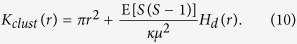


The density of the point pattern will be *μκ*. When 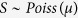, since *E*[*S*(*S* − 1)] = *μ*^2^ for Poisson distribution, (10) reduces to


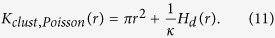


The derivation in case of other distributions for points per cluster is straightforward. In the case of geometric or exponential distribution of *S*, behavior often observed in nanoimaging^45^,^46^,^47^, 
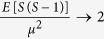
 for 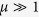 Thus, for a large class of cluster models, the *K*-function is independent of the number of molecules per cluster *µ*.

Note that *H*_*d*_, being the CDF, is monotonic and non-decreasing. The corresponding PCF 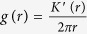 becomes:


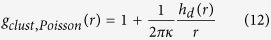


The PCF and *K*-function for different cluster shapes are given in Supplementary Table S1, and the shapes of their PDFs are given in Supplementary Information. Note that disk clusters contain points distributed uniformly at random within a circle (disk), a process known as Matérn cluster process in spatial statistics (the case of Gaussian cluster shapes is known as Thomas process). Also note that *r*_*t*_ is defined differently for different cluster models: for a disk cluster, *r*_*t*_ = *R*, the true cluster radius, whereas for Gaussian clusters, we set *r*_*t*_ = *σ*, the true standard deviation (the full list can be found in Supplementary Table S1). We also add the physical Ising model to the compilation, since it is one of the models that has been proposed for membrane protein clustering^18^, even though it is not a Neyman-Scott process. Also, note that the exponential approximation *g*_*a*_(*r*) has the same shape as the variance Gamma function model (varGamma) in Supplementary Table S1, pointing at the non-uniqueness of *g*(*r*) shapes and the difficulty of identifying cluster models from data based on their PCF shapes.

### Effect of background

To model a monomer fraction or background, a spatial Poisson distributed monomer point pattern can be superimposed to a purely clustered process, such that the purely clustered fraction of points is *β*. The resulting *K*-function and PCF can be obtained using the expression for superposition of two independent point processes^41^. In the case of a clustered process with *g*(*r*) = 1 + *Bv*(*r*), superposition with such a background process results in the PCF:


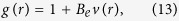


where *B*_*e*_ = *Bβ*^2^, *β* being the purely clustered fraction^41^. Expressions for *K*(*r*) and *L*(*r*) − *r* undergo similar scaling in parameter. It can be noted that the shape of the function remains the same as the purely clustered process, the change in parameter *B* being the only change, again pointing at the non-uniqueness of PCF shapes, and the quadratic effect of background on the function (note the effect on (7)).

### Simulation and analysis details

All simulations were done in R, using the spatstat library^48^. Simulations of cluster processes were done with standard library functions, such as rThomas and rMatClust. Parameter estimation by minimum contrast method was done using kppm function, and using parameters “Thomas” and “VarGamma”. Analytical derivations were performed with the help of symbolic algebra software [Mathematica(Wolfram Research, USA)].

## Additional Information

**How to cite this article**: Shivanandan, A. *et al.* On characterizing protein spatial clusters with correlation approaches. *Sci. Rep.* **6**, 31164; doi: 10.1038/srep31164 (2016).

## Supplementary Material

Supplementary Information

## Figures and Tables

**Figure 1:**
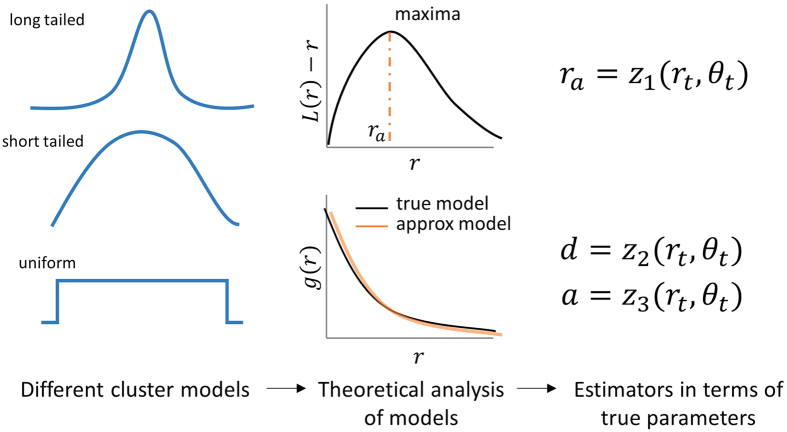
Theoretical relations between estimators and true parameters for different cluster models. Cartoon figures that elucidate our approach show different cluster models (left), each for which the theoretical correlation functions *L*(*r*) − *r* and pair correlation function *g*(*r*) were derived. Then the theoretical expressions are obtained for the estimators considered: the radius of maximal aggregation *r*_*a*_ (middle, top) and estimation based on fitting to an exponential functional approximation for the PCF (middle, bottom). From these, the relation between the estimators and the true parameters of the models are derived (right). *r*_*t*_ is true radius parameter, *θ*_*t*_ denotes other true parameters, e.g., number of clusters per unit area. For the cluster models (left), shown are projections of 2D distributions, one with heavy concentration at the center (top) and another with a smoother distribution (middle), followed by uniform distribution (bottom).

**Table 1:** Theoretical scaling for different cluster models, in using the exponential approximation for PCF and using Least Square Error criteria.

Cluster model	*m* = *d*/*r*_*t*_ *d*,*r*: cluster size	*n* = *a*/*a*_*t*_ *a*: amplitude	*l* = *N*_*a*_/*N*_*t*_ *N*: molecules per cluster
Gaussian	1.54	1.26	1.48
disk	0.8	1.81	1.48
Cauchy	1.7	1.17	0.85
varGamma	1	1	1
Ising	0.5	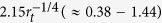	0.59

*d*, *a*, *N*_*a*_ correspond to the approximate PCF model *g*_*a*_(*r*) = 1 + *a* exp(−*r*/*d*). True parameters *r*_*t*_, *a*_*t*_ and *N*_*t*_ corresponding to the model PCFs of the form *f*(*r*) = *a*_*t*_*v*(*r*, *r*_*t*_) can be obtained from Supplementary Table S1 and using (7). The minimum *r*_*m*_ value, used in the calculation of the Squared Error *E* in (3), for each model is as follows: Gaussian - 6*σ*, disk - 3*R* and Ising - 4*ξ*, and higher values for *r*_*m*_ give the same results. In the case of Cauchy model *r*_*m*_ = ∞ was used, and for varGamma any *r*_*m*_ > 0 corresponds to the results in the table, since the PCF shapes match perfectly. The *m*_0.95_ values: *m*_0.95_ = *d*/*r*_0.95_, *r*_0.95_ being the scale at which 95% of points are expected to lie, are 0.63, 0.82, 0.38 and 0.28 respectively, for Gaussian, disk, Cauchy and varGamma models.
