# Supplementary material for: On characterizing protein spatial clusters with correlation approaches
Source: Sci Rep. 2016 Aug 10;6:31164. doi: 10.1038/srep31164 (PMC4979030; doi:10.1038/srep31164)
Supplement: Supplementary Information [file srep31164-s1.pdf]

Supplementary Information for *On characterizing protein spatial clusters with correlation approaches*

A. Shivanandan, J. Unnikrishnan, A. Radenovic

Supplementary Notes Contents

1 Derivation of expressions for $p = \frac{r_a}{r_t}$ . . . 2
2 95% radius for different models . . . 2
3 Proofs regarding lower bound for radius of maximal aggregation . . . 2
4 Radius of maximal aggregation in the case of $\tilde{K}(r, n)$ of Lagache et al . . . 3
5 Bias in parameter estimation based on exponential PCF approximation . . . 4
6 Case of power law PCF . . . 5

Supplementary Figures List

S1 Maximal aggregation: Comparison of $p = r_a/r_t$ from theory and simulations, with errorbars 6
S2 Scaling in exponential PCF approach: d/D for Ising model . . . 6
S3 Scaling in exponential PCF approach: a/A for Ising model, its dependency on D . . . 7
S4 Exponential PCF approach: comparison between theory and simulations, with errorbars . . 7
S5 Exponential PCF approach, with a power law true PCF . . . 8

Supplementary Tables List

S1 Cluster models used for analysis. . . . 9
S2 Exact expressions for the radius of maximal aggregation r_a for different cluster models. . . 9

Supplementary Notes

1 Derivation of expressions for $p = \frac{r_a}{r_t}$

Here we derive the relation in the case of Neyman-Scott process with Gaussian shaped clusters. The derivation in the case of other distributions are similar, starting from the expressions in Supplementary Table S1.

We start from the K-function for Gaussian shaped clusters:

$$K(r) = \pi r^2 + \frac{1}{\kappa}(1 - \exp(\frac{-r^2}{4\sigma^2})). \tag{1}$$

In the form $K(r) = \pi r^2 + \frac{1}{A}H(r)$ as in Main Text, this corresponds to $A = \kappa$,$H(r) = 1 - \exp(\frac{-r^2}{4\sigma^2})$ and $h(r) = \frac{r}{2\sigma^2}\exp(\frac{-r^2}{4\sigma^2})$. Substituting in the equation

$$A = \frac{h(r_a)^2}{4\pi(H(r_a) - r_a h(r_a))} \tag{2}$$

from Main Text and rearrangement will give the relation as in Supplementary Table S2.

2 95% radius for different models

These were found by solving the CDF $\int_0^r f_{pdf}(r)dr = .95$ for r, where $f_{pdf}(r)$ is the radial probability density function for each model(1–3). In the case of Cauchy and varGamma models, marginal PDFs of r in polar coordinates were obtained from the bivariate PDFs in cartesian coordinates by standard transformation(multiplication by $2\pi r$). The results are given in the following table, along with the 95% limits. $K_\nu(.)$ denotes the modified Bessel function of the second kind.

Model	$f_{pdf}(r)$	$r_{.95} = u_{.95}r_t$	Lower bound for $p_{.95}$
Gaussian	$\frac{r}{\sigma^2}\exp\left(-\frac{r^2}{2\sigma^2}\right)$	2.448σ	.914
disk	$\frac{2r}{R^2}$	$.975R$	1.329
Cauchy	$\frac{r}{\omega^2}\left(1 + \frac{r^2}{\omega^2}\right)^{-3/2}$	4.469ω	.568
VarGamma	$\frac{\sqrt[4]{2}r^{3/4}K_{-\frac{1}{4}}\left(\frac{r}{\eta}\right)}{\eta^{7/4}\Gamma\left(\frac{3}{4}\right)}$	3.547η	.505

3 Proofs regarding lower bound for radius of maximal aggregation

Lemma .1. *Let $h : \Re^+ \mapsto \Re^+$ be a unimodal differentiable function with a unique maximum at $r_m > 0$ and a derivative satisfying $h'(r) > 0$ for $0 \le r < r_m$, and $h'(r) < 0$ for $r > r_m$. Note: this is satisfied by all the models in Supplementary Table S1.*

Further assume that there exists $r^* > 0$ that satisfies

$$H(r^*) - r^* h(r^*) = 0.$$

Then the radius of maximal aggregation $r_a \ge r^*$ where r_a is obtained as a solution to (2) for some $A > 0$.

Furthermore as $A \to \infty$, we have $r_a \to r^*$.

Proof. Define

$$w(r) = H(r) - rh(r).$$

Clearly $w(0) = 0$ and the derivative satisfies $w'(r) = -rh'(r)$.

From the properties of h' we have $w'(r) \le 0$ for $0 \le r < r_m$, with strict inequality for $0 < r < r_m$, and $w'(r) > 0$ for $r > r_m$. Hence

$$w(r) < 0 \text{ for } 0 < r \le r_m. \tag{3}$$

Since $w(r^*) = 0$ it follows that $r^* > r_m$. Moreover since $w'(r)$ is strictly positive for $r \in (r_m, r^*]$, it follows that $w(r) < 0$ for $r \in (r_m, r^*)$. Combining with (3) it follows that $w(r) < 0$ for $r \in (0, r^*)$.

Now, we know that r_a satisfies (2) for some $A > 0$. Thus we must have $w(r_a) > 0$ and hence it follows that $r_a \geq r^*$.

Now consider the situation in which $A \to \infty$. Define

$$z(r) = \frac{h(r)^2}{H(r) - rh(r)}$$

to denote the expression on the right hand side of (2) without the factor of 4π included. Since $z(r) = \frac{h(r)^2}{w(r)}$ we know from the earlier analysis of w that $z(r) \leq 0$ for $r < r^*$ and $z(r) \geq 0$ for $r < r^*$. Now consider the derivative of z. We have

$$\begin{aligned} z'(r) &= \frac{(H(r) - rh(r))2h(r)h'(r) + rh'(r)h(r)^2}{(H(r) - rh(r))^2} \\ &= \frac{2h(r)h'(r)H(r) - rh'(r)h(r)^2}{(H(r) - rh(r))^2} \\ &= \frac{h(r)h'(r)(2H(r) - rh(r))}{(H(r) - rh(r))^2} \end{aligned} \quad (4)$$

Now consider the function $q(r) = 2H(r) - rh(r)$ for $r \geq r^*$. At $r = r^*$ we have $q(r^*) = 2H(r^*) - r^*h(r^*) = H(r^*) > 0$. Moreover the derivative of this function is $q'(r) = h(r) - rh'(r)$ which is non-negative for $r > r^*$ because $h'(r) < 0$. Thus $q(r) > 0$ for $r > r^*$. This observation combined with the fact that $h'(r) < 0$ for $r > r^*$ and (4) implies that $z'(r) < 0$ for $r > r^*$. Thus we have that z is strictly decreasing in the interval (r^*, ∞). Moreover $z(r) \to \infty$ as r approaches r^* from above. Hence as $A \to \infty$ the left hand side of (2) $\to \infty$ and thus by virtue of (2) we must have $r_a \to r^*$. □

4 Radius of maximal aggregation in the case of $\tilde{K}(r, n)$ of Lagache et al

Setting $\frac{\partial \tilde{K}(r,n)}{\partial r} = 0$ for disk clusters as discussed in Main Text, followed by routine manipulations lead us to the relation:

$$\begin{aligned} &-\frac{0.0210642p^2\left(\left(16-4p^2\right)\cos^{-1}(0.5p) + p\sqrt{4-p^2}\left(p^2-4\right)\right)\left(6.0286m^3 + 7.35489m^2p - 18.9394mp^2 + np^3\right)}{p^2-4} \\ &+ 0.00789906p\left(2.45163m^2 - 12.6263mp + np^2\right)\left(\sqrt{4-p^2}\left(p^2+2\right)p - 8p^2\cos^{-1}\left(\frac{p}{2}\right) - 8\sin^{-1}\left(\frac{p}{2}\right)\right) \\ &+0.0317468\left(m^3 + 1.22m^2p - 3.14159mp^2 + 0.165876np^3\right)\left(\sqrt{4-p^2}\left(p^2+2\right)p - 8p^2\cos^{-1}\left(\frac{p}{2}\right) - 8\sin^{-1}\left(\frac{p}{2}\right)\right) \\ &= 0, \end{aligned} \quad (5)$$

where $p = \tilde{r}_a/R$, $m = side/R$ where $A = side^2, P = 4.side$.

The contour plot of p vs m, based on this expression, is shown in the Main Text, for different values of n.

In the case of Gaussian clusters, the relation is simpler:

$$\begin{aligned} m^3\left(p^2 - 2e^{\frac{p^2}{4}} + 2\right) + m^2p\left(1.22p^2 - 3.66e^{\frac{p^2}{4}} + 3.66\right) + mp^2\left(-3.14159p^2 + 12.5664e^{\frac{p^2}{4}} - 12.5664\right) \\ + np^3\left(0.165876p^2 - 0.82938e^{\frac{p^2}{4}} + 0.82938\right) = 0, \end{aligned} \quad (6)$$

and the corresponding contour plot is provided in Main Text.

5 Bias in parameter estimation based on exponential PCF approximation

We simply show the case for Ising model. Derivation for other models follow the same procedure. For $g_a(r) = 1 + a\exp(-r/d)$ and $f(r) = 1 + Ar^{-1/4}\exp(-r/D)$, the Least Squared Error criteria gives:

$$(\hat{a}, \hat{d}) = \arg\min_{a,d} E = \arg\min_{a,d} \int_0^{r_m} (f(r) - g_a(r))^2 dr. \tag{7}$$

We obtain: $E = -\frac{1}{2}a^2 d\left(-1 + e^{-\frac{2r_m}{d}}\right) + \frac{A^2\sqrt{\frac{\pi}{2}}\sqrt{r_m}\mathrm{Erf}\left[\sqrt{2}\sqrt{\frac{r_m}{D}}\right]}{\sqrt{\frac{r_m}{D}}} - \frac{2aAr_m^{3/4}\left(\Gamma\left[\frac{3}{4}\right]-\Gamma\left[\frac{3}{4},\frac{(d+D)r_m}{dD}\right]\right)}{\left(\frac{(d+D)r_m}{dD}\right)^{3/4}}$

$$\frac{\partial E}{\partial a} = 0 \implies \frac{\partial E}{\partial a} = -ad\left(-1 + e^{-\frac{2r_m}{d}}\right) - \frac{2Ar_m^{3/4}\left(\Gamma\left[\frac{3}{4}\right]-\Gamma\left[\frac{3}{4},\frac{(d+D)r_m}{dD}\right]\right)}{\left(\frac{(d+D)r_m}{dD}\right)^{3/4}}=0$$

$$\frac{\partial E}{\partial d} = 0 \implies \frac{\partial E}{\partial d} = -\frac{1}{2}a^2\left(-1 + e^{-\frac{2r_m}{d}}\right) - \frac{a^2 e^{-\frac{2r_m}{d}} r_m}{d} - \frac{2aAdDe^{-\frac{(d+D)r_m}{dD}}\left(\frac{r_m}{dD} - \frac{(d+D)r_m}{d^2D}\right)}{(d+D)r_m^{1/4}} + \frac{3aAr_m^{3/4}\left(\frac{r_m}{dD} - \frac{(d+D)r_m}{d^2D}\right)\left(\Gamma\left[\frac{3}{4}\right]-\Gamma\left[\frac{3}{4},\frac{(d+D)r_m}{dD}\right]\right)}{2\left(\frac{(d+D)r_m}{dD}\right)^{7/4}}=0$$

Solving both equations separately for $a = \hat{a}$, we obtain:

$$\hat{a} = \frac{2Ae^{\frac{2r_m}{d}} r_m^{3/4}\left(\Gamma\left[\frac{3}{4}\right]-\Gamma\left[\frac{3}{4},\frac{(d+D)r_m}{dD}\right]\right)}{d\left(-1+e^{\frac{2r_m}{d}}\right)\left(\frac{(d+D)r_m}{dD}\right)^{3/4}}$$

and,

$$\hat{a} = \frac{\frac{4ADe^{-\frac{(d+D)r_m}{dD}} r_m^{3/4}}{d(d+D)} - \frac{3Ar_m^{7/4}\Gamma\left[\frac{3}{4}\right]}{d^2\left(\frac{(d+D)r_m}{dD}\right)^{7/4}} + \frac{3Ar_m^{7/4}\Gamma\left[\frac{3}{4},\frac{(d+D)r_m}{dD}\right]}{d^2\left(\frac{(d+D)r_m}{dD}\right)^{7/4}}}{-1+e^{-\frac{2r_m}{d}} + \frac{2e^{-\frac{2r_m}{d}} r_m}{d}}$$

Equating both the above expressions of $\hat{a}$, simplifying, and setting $m = d/D$ and $k = r_m/D$, we get:

$$\frac{2e^{\frac{2k}{m}}\left(\Gamma\left(\frac{3}{4}\right)-\Gamma\left(\frac{3}{4},k\left(1+\frac{1}{m}\right)\right)\right)}{e^{\frac{2k}{m}}-1} + \frac{me^{k\left(\frac{1}{m}-1\right)}\left(4\left(\frac{k}{m}+k\right)^{3/4}-3\Gamma\left(\frac{3}{4}\right)e^{\frac{k}{m}+k}+3e^{\frac{k}{m}+k}\Gamma\left(\frac{3}{4},k\left(1+\frac{1}{m}\right)\right)\right)}{(m+1)\left(m\left(e^{\frac{2k}{m}}-1\right)-2k\right)} = 0$$

Note that this equation does not contain the amplitude parameters a and A. A contour plot of this equation is shown in Supplementary Figure S2. For reasonably large values of r_m (i.e., $r_m > 2D$), $m = \hat{d}/D = .5$. That is, the correlation length parameter estimated by the approximate model is half of the correlation length of the true model.

From these results, the parameter values $k = 4, m = .5$ (or any $k > 2$) can be substituted in the expression for $\hat{a}$, to obtain:

$$n = \frac{a}{A} = 2.15031D^{-1/4}$$

That is, the amplitude parameter of the approximate model is dependent on both the true amplitude parameter as well as the correlation length. The relationship is shown in Supplementary Figure S3. This parameter could be $n = .38 - 1.44$ scaled from the true amplitude parameter for $D = 5 - 1000nm$, relevant scales for membrane protein clusters.

Now, the average number of points per cluster:

$$N_I = 1 + \rho\int_0^{\infty}(f(r) - 1)2\pi r dr \approx 2\pi AD^{1.75}\Gamma\left(\frac{7}{4}\right)$$

$$N_a \approx 2\pi ad^2\rho = 3.3777AD^{1.75} = 0.584919N_I$$

That is, the approximate model underestimates the average number of points per cluster by over 40%.

6 Case of power law PCF

In the case of the PCF $g(r) = 1 + c\left(\frac{r_0}{r}\right)^s$, assuming $s \neq 1$,

$$K(r) = \pi r^2 + \frac{2\pi c}{2-s}\left(\frac{r_0}{r}\right)^s r^2 \tag{8}$$

for $s < 2$.

A in (10) of Main Text will be $A = \frac{2-s}{2\pi c}$. Using (10), we get:

$$p = \frac{r_a}{r_0} = \left(\frac{c(2-s)}{2(s-1)}\right)^{1/s}. \tag{9}$$

A plot of this equation for different s is shown in Supplementary Figure S5. It can be seen that p varies across orders of magnitude based on values of s and c.

Supplementary Figures

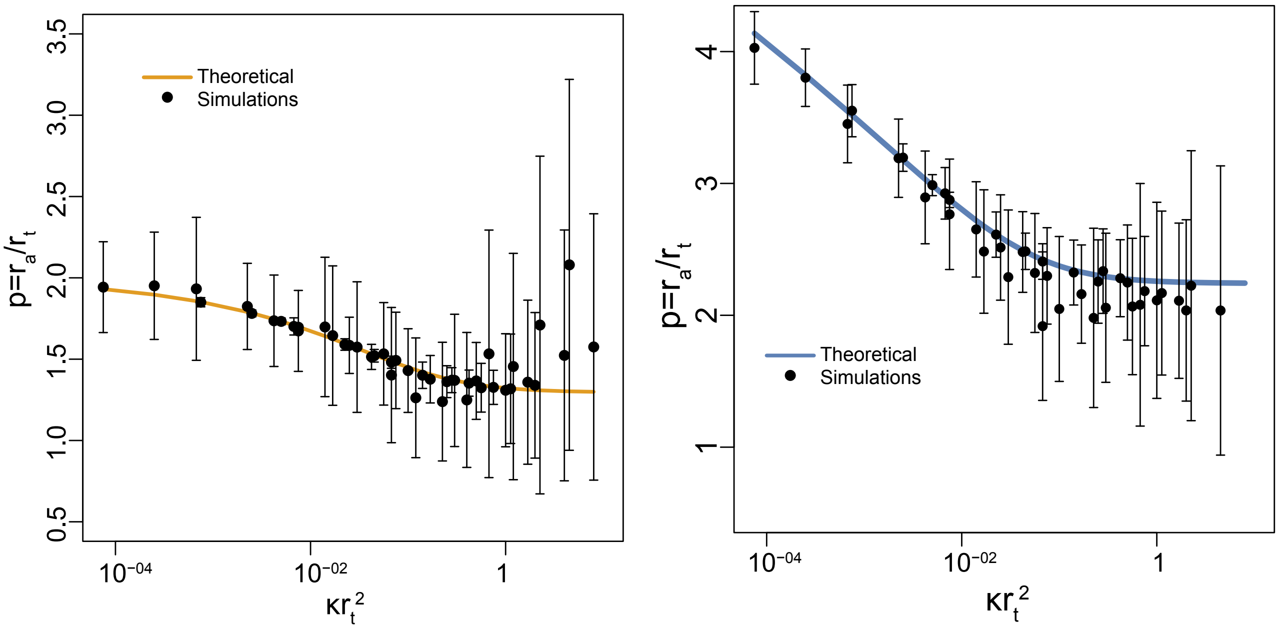

Supplementary Figure S1: Comparison of $p = r_a/r_t$ from theory and simulations. Figure 2 in Main Text with error bars(σ).

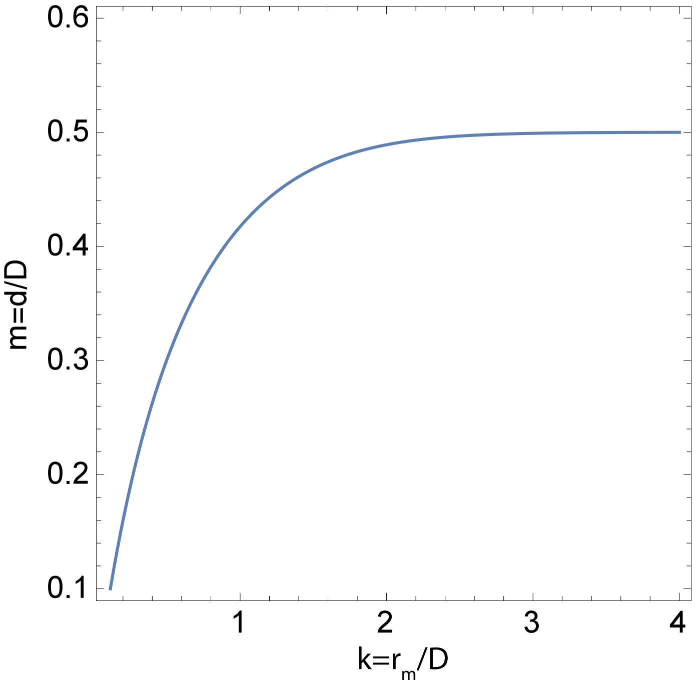

Supplementary Figure S2: Contour plot of $k = r_m/D$ vs $m = d/D$ for Ising model. r_m is the distance value to which the Least Squares sum is taken, where D is the true size parameter of the Ising model, and d that of the exponential approximation of PCF. After $\approx r_m > 2D$, the m value is fixed at .5.

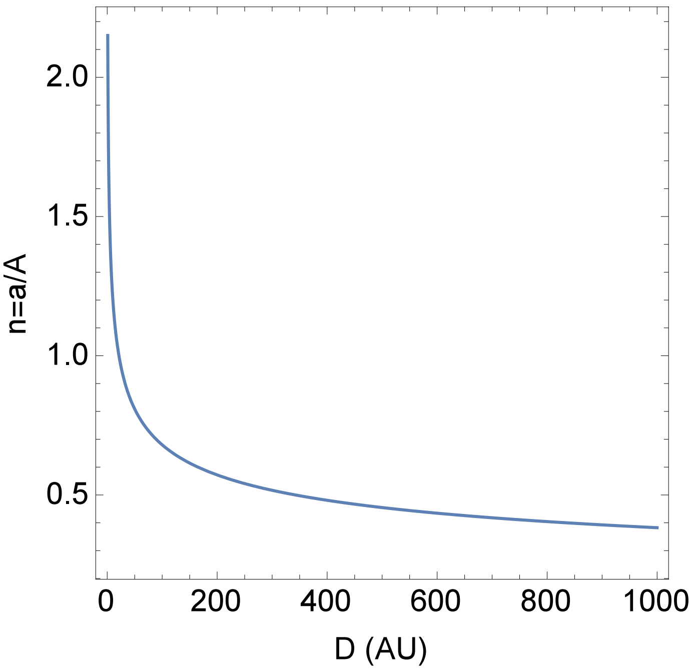

Supplementary Figure S3: Plot of D vs $n = a/A$ for Ising model, at $k = 4, m = .5$. See Supplementary Figure S2 for details on parameteric values of k, m.

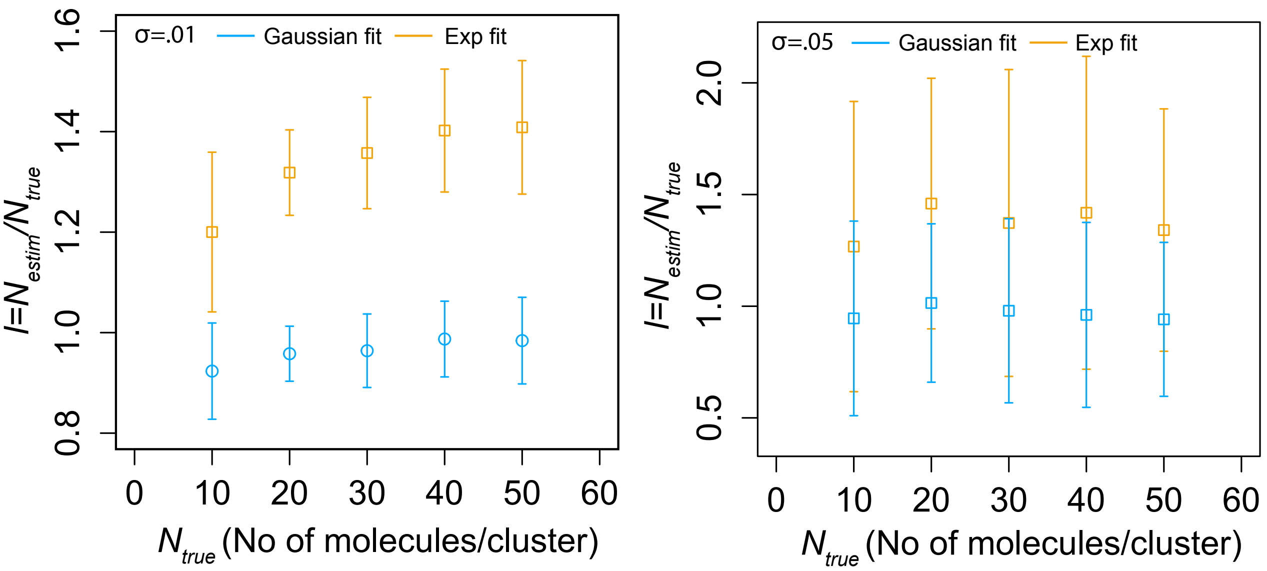

Supplementary Figure S4: Comparison of fitting empirical PCF of Gaussian clusters to (1) exponential PCF g_a and (2) theoretical PCF of Gaussian clusters, for different true cluster σ. Figure 6b in Main Text shown with error bars(σ).

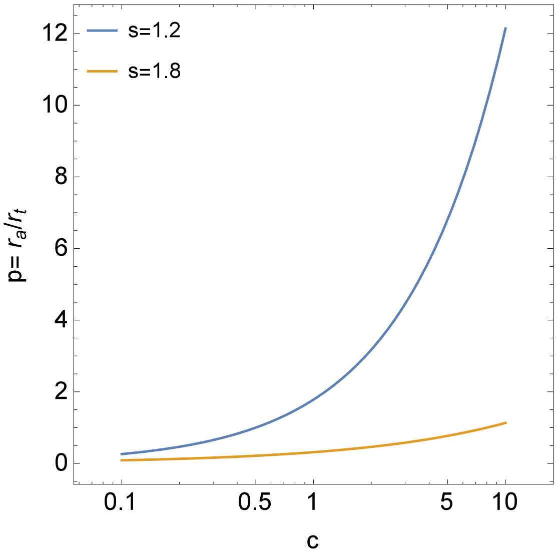

Supplementary Figure S5: Ratio of radius of maximal aggregation to true cluster size parameter $p = \frac{r_a}{r_0}$ for power law PCF, as a function of amplitude parameter c for different values of power s. Depending on s, p could be crucially dependent on c.

Supplementary Tables

Model (r_t)	$g(r)-1$	$K(r)-\pi r^2$
Gaussian (σ) (1)	$\frac{1}{4\pi\kappa\sigma^2}\exp(\frac{-r^2}{4\sigma^2})$	$\frac{1}{\kappa}(1-\exp(\frac{-r^2}{4\sigma^2}))$
disk (R) (1)	$\frac{2}{\pi^2 R^2\kappa}(\cos^{-1}(\frac{r}{2R})-\frac{r}{2R}\sqrt{1-\frac{r^2}{4R^2}})$	†
Cauchy (ω)(2)	$\frac{1}{8\pi\omega^2\kappa}(1+\frac{r^2}{4\omega^2})^{-3/2}$	$\frac{1}{\kappa}(1-\frac{1}{\sqrt{1+\frac{r^2}{4\omega^2}}})$
variance Gamma $\nu=1/2$ (η) (3)	$\frac{1}{2\pi\eta^2\kappa}\exp(-r/\eta)$	$\frac{1}{\kappa}\left(1-e^{-\frac{r}{\eta}}\left(1+\frac{r}{\eta}\right)\right)$
Ising (4)	$a_I r^{-1/4}\exp(-r/\xi)$	$2\pi a_I\xi^{7/4}\left(\Gamma\left(\frac{7}{4}-\Gamma\left(\frac{7}{4},\frac{r}{\xi}\right)\right)\right)$

Supplementary Table S1: **Cluster models used for analysis.**† $\frac{2}{\kappa\pi}(\frac{r^2\cos^{-1}\left(\frac{r}{2R}\right)}{R^2}-\frac{r\sqrt{1-\frac{r^2}{4R^2}}\left(r^2+2R^2\right)}{4R^3}+\sin^{-1}\left(\frac{r}{2R}\right))$. Also, for disk model, the functions provided here are for $r\leq 2R$, for $r>2R$, it is 0. Note that for disk, $g(r)=1$ at $r\geq 2R$, which provides a simple estimator for R.

Cluster model	Expression for $p=r_a/r_t$	Theoretical lower bound for p (to 5 digits)
Gaussian ($p=r_a/\sigma$)	$\kappa\sigma^2=\frac{e^{-\frac{p^2}{4}}p^2}{8\pi\left(-p^2+2e^{\frac{p^2}{4}}-2\right)}$	2.24181
Disk ($p=r_a/R$)	$\kappa R^2=\frac{p^2\left(p\sqrt{4-p^2}-4\arccos\left(\frac{p}{2}\right)\right)^2}{\pi^2\left(\sqrt{4-p^2}(3p^2-2)p-8p^2\arccos\left(\frac{p}{2}\right)+8\arcsin\left(\frac{p}{2}\right)\right)}$	1.29564
Cauchy($p=r_a/\omega$)	$\kappa\omega^2=\frac{p^2}{\pi(p^2+4)^{3/2}\left((p^2+4)^{3/2}-4p^2-8\right)}$	2.54404
varGamma ($p=r_a/\eta$)	$\kappa\eta^2=\frac{p^2}{4\pi(\exp(2p)-\exp(p)(p^2+p+1))}$	1.79328
Ising ($p=r_a/\xi$)	$\frac{1}{2\pi}a_I^{-1}\xi^{1/4}=\frac{\exp(-2p)p^{3/2}}{4\pi\left(-\exp(-p)p^{7/4}-\Gamma\left(\frac{7}{4},p\right)+\Gamma\left(\frac{7}{4}\right)\right)}$	1.37220

Supplementary Table S2: **Exact expressions for the radius of maximal aggregation r_a for different cluster models.**

References

[1] Illian, J., Penttinen, A., Stoyan, H. & Stoyan, D. *Statistical analysis and modelling of spatial point patterns*, vol. 70 (John Wiley & Sons, 2008).

[2] Ghorbani, M. Cauchy cluster process. *Metrika* **76**, 697–706 (2013).

[3] Jalilian, A., Guan, Y. & Waagepetersen, R. Decomposition of Variance for Spatial Cox Processes. *Scand J Stat* **40**, 119–137 (2013).

[4] Veatch, S. L. *et al.* Correlation functions quantify super-resolution images and estimate apparent clustering due to over-counting. *PLOS ONE* **7**, e31457 (2012).
